# Enhanced memory and hippocampal connectivity in humans 2 days after brief resistance exercise

**DOI:** 10.1002/brb3.3436

**Published:** 2024-02-21

**Authors:** Teruo Hashimoto, Rikimasa Hotta, Ryuta Kawashima

**Affiliations:** ^1^ Department of Functional Brain Imaging, Institute Development, Aging and Cancer Tohoku University Sendai Japan; ^2^ Tohoku University School of Medicine Sendai Miyagi Japan

**Keywords:** brain plasticity, fMRI, muscles, strength training

## Abstract

**Introduction:**

Exercise has significant health benefits and can enhance learning. A single bout of high‐intensity resistance training may be sufficient to improve memory. This study aimed to assess memory enhancement by a single bout of high‐intensity resistance training and to examine the neural underpinnings using resting‐state functional magnetic resonance imaging (MRI).

**Methods:**

Sixty young adults (34 men and 26 women), divided into the training and control groups, participated. The first session included verbal memory recall tests (cued‐ and free‐recall), resting‐state functional MRI (rs‐fMRI), and a single‐bout high‐intensity resistance training for the training group. Two days later, they underwent post‐intervention memory tests and rs‐fMRI. The study design was 2 groups × 2 sessions for memory tests, and within training group comparisons for rs‐fMRI.

**Results:**

Compared to the control group without resistance training, the training group showed higher cued‐recall performance 2 days after the brief resistance training (training: +0.27, control: −0.13, interaction: *p* = .01), and their free‐recall scores were associated with enhanced left posterior hippocampal connectivity (*r* = .64, *p* < .001).

**Conclusions:**

These results suggest that brief high‐intensity resistance exercise/strength training could enhance memory without repeated exercising. The quick effect of resistance training on memory and hippocampal connectivity could be revealed. A focused and one‐shot exercise may be sufficient to enhance memory performance and neural plasticity in a few days.

## Graphical abstract

A single bout high‐intensity resistance training improved memory in young adults. Higher recall scores were observed two days after the resistance training. The recall scores were related to enhanced left posterior hippocampal connectivity.

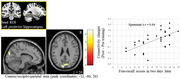

## 1 LIMITATIONS

Cued‐recall tests that were originally made for brain‐damaged patients were too easy for our participants and could not be suitable to examine effects of interventions in healthy young adults. However, slightly reduced cued‐recall score in the second session in the control group might suggest more proactive interference from the first session than in the training group. We must emphasize that causal relations cannot be examined by our correlational analyses; however, we speculate about them from our results. Although a high stability of rsFC between sessions in 10 different days has been shown (Gratton et al., 2018), the absence of FC data in the control group without resistance training in this study could limit the interpretation of our results. It might be possible that participants in the training group are more engaged in aerobic training that enhances hippocampal connectivity than those in the control group (Stillman et al., 2018; Suwabe et al., 2018; Tozzi et al., 2016). In addition, alcohol/caffeine consumption and sleep duration, which can negatively influence the hippocampus and neuroplasticity (Loheswaran et al., 2017; Walker et al., 2021), might be different among groups. Moreover, if memory tests could modulate hippocampal FC, FC changes in the training group could not be attributed to resistance training. Some participants showed reduced connectivity after 2 days of training (<0 on the *y*‐axis of the scatter plot in Figure 4). Further studies are necessary to determine the cause of these individual differences. Some individuals may require more time (e.g., 1 more day of interval) to detect the effects of muscle training. A few participants could not complete their training set, which could reduce the effects of muscle training. Alternatively, some individuals may not benefit greatly in memory performance following muscle training. Finally, our results from young adults might not be applicable to the other age groups.

## 2 INTRODUCTION

Several strategies for improving learning and memory have been explored, such as elaborative encoding and retrieval practices. Additionally, caloric restriction (Hashimoto & Watanabe, 2005; Witte et al., 2009) and physical exercise (Roig et al., 2013; Stillman et al., 2020) improve memory. Both aerobic and resistance exercises have demonstrated beneficial effects on memory with neuroplasticity (Aghjayan et al., 2021; Voss et al., 2019), whereas more studies are required to elucidate the effects of resistance training on memory (Loprinzi et al., 2021; Mendelski et al., 2024; Pontifex et al., 2009; Venezia et al., 2023; Weinberg et al., 2014; Wu et al., 2021).

Resistance training is a kind of muscle‐strengthening activity that causes the body's muscles to work or hold against an applied force or weight (Services, 2018), and that is effective in enhancing several aspects of physical and mental health in adults of all ages (Westcott, 2012). Moderate‐to‐high‐intensity resistance muscle training improves physical, metabolic, and psychological health in older adults (Fragala et al., 2019). Insufficient intensity of resistance training in older adults can result in inconsistent benefits on memory (Loprinzi et al., 2018). Besides age, several other factors, including individual differences, may vary the effects of exercise on memory (Loprinzi, 2019). Nonetheless, a single bout of high‐intensity resistance training reportedly enhances visual recognition memory 48 h later in young adults (Weinberg et al., 2014).

In rodents, neuroplasticity in the hippocampus was shown to be a mechanism by which memory improves with a single bout of high‐intensity resistance training (Fernandes et al., 2016). Resistance training improves memory by inducing insulin growth factor and modulating neuroplasticity and inflammatory factors, such as protein kinase C, tumor necrosis factor alpha (TNF‐α), and interleukin‐1 beta (IL‐1β) (Cassilhas et al., 2016; Vilela et al., 2017). Similarly, protein kinase A, TNF‐α, and IL‐1β increase with resistance training (Vilela et al., 2017). Furthermore, resistance training increases the levels of hippocampal brain–derived neurotrophic factor, tyrosine‐related kinase B, and cyclic adenosine monophosphate response element‐binding protein (Lee et al., 2012). In humans, the positive effects of aerobic exercise on memory and the hippocampus are highly consistent (Aghjayan et al., 2021); however, benefits of resistance training have been less investigated.

Resting‐state functional connectivity (rsFC), which is robust across different days and task/rest states, is useful for detecting individual characteristics of the brain network (Gratton et al., 2018). Aerobic exercise enhances hippocampal FC in young adults (Stillman et al., 2018; Tozzi et al., 2016). A 10‐min mild exercise program improves both hippocampal connectivity and recognition of visual objects (Suwabe et al., 2018). High‐intensity resistance training may enhance the hippocampal FC, which may be associated with memory improvement.

This study aimed to examine the acute effects of high‐intensity resistance training on memory and hippocampal FC. We used standardized verbal recall tests to examine memory performance, and a 2‐day delay in memory tests was used according to a previous study demonstrating memory enhancement by a single bout of resistance training (Weinberg et al., 2014). We hypothesized that the hippocampal network can be enhanced by brief high‐intensity resistance training and that an enhanced network is associated with better memory performance.

## 3 METHODS

### 3.1 Participants

Sixty healthy young adults (34 men and 26 women) aged 19–27 years (mean = 21.5) not engaged in self‐reported habitual muscle training (more than once a week) participated. All participants were undergraduate/postgraduate students at Tohoku University recruited via the Tohoku University student career center. They were randomly divided into the training group (18 men and 12 women) and the control group (16 men and 14 women). Participants in the training group engaged in a muscle strength training session on the first day (intervention), and the second session 2 days later (range: 48–51 h; post‐intervention). Participants in the control/waiting group engaged in two sessions without muscle training. All participants were not given any instructions about food intake, alcohol/caffeine consumption, and sleep duration. They were inhibited to engage in muscle training before the second session. Their levels of aerobic activity were not measured. This study was approved by the local ethics committee of the Tohoku University Smart Aging Center (2109‐01). The study was conducted in accordance with the Declaration of Helsinki, and written informed consent was obtained from all participants.

### 3.2 Experimental procedures

**FIGURE 1:**
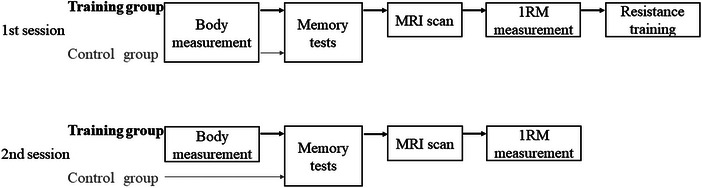
Experimental protocol. Procedures in the pre‐intervention on the first session were shown in the upside, and those in the post‐intervention on 2 days after the first session (second session) were shown in the bottom. 1RM, one repetition maximum.

On the intervention day, participants’ heights and body weights were measured. Subsequently, two memory tests were conducted for both the training and control groups. Magnetic resonance imaging (MRI) with a duration of 15 min was performed immediately after the memory tests only for the training group. Finally, muscle strength measurements and resistance muscle training were performed in predetermined order for the training group. The interval between memory tests and muscle training was approximately 20 min because more memory enhancement may be expected if exercise is timed in close temporal proximity to the learning/encoding session (Roig et al., 2016). Resistance training (only on the intervention day), MRI, and all measurements during both sessions were conducted between 1 and 4 p.m. Only two memory tests were conducted for the control group in the first session (Figure 1).

Post‐intervention session included body weight measurement, two memory tests, muscle strength measurement, and MRI, performed in predetermined order for the training group. Only two memory tests were conducted for the control group in the post‐intervention session.

#### 3.2.1 Height and body weight measurements

Height and body weight were measured (BWB‐800, TANITA Inc.) during both the sessions, and body mass index (BMI) was calculated.

#### 3.2.2 Memory tests

Memory tests were identical in the training and control groups. Two verbal recall memory tests were used in the order: the auditory verbal learning test (AVLT) followed by standard verbal paired‐associate learning test (PA). In the training group, these two memory tests were performed before muscle training during the intervention session and before muscle strength measurement in the post‐test.

AVLT is a free recall test consisting of 15 words, including both immediate and delayed recall assessments (Lezak et al., 2012). During the intervention, before muscle training, participants were asked to recall a list of words (list A) spoken by an experimenter. After the 5 immediate recall assessments, participants were asked to listen to and recall another list of 15 words (list B). Subsequently, the participants were asked to recall the first list of words (list A, short‐term delayed recall). The participants were not instructed on the number of words in the list, even if they asked for it.

PA is a cued‐recall test consisting of 6 lists of 10 paired words, originally for patients with brain damage (Japan Society for Higher Brain Dysfunction, 2014). Half of the list (A1, B1, and C1) consists of semantically related paired words (e.g., stamp‐postcard), and the other half (A2, B2, and C2) consists of semantically unrelated paired words (e.g., lipstick‐river). Related and unrelated lists (A1 and A2) were used in the first session. Participants were asked to listen to 10 paired words and recall each word paired to cue a word (e.g., postcard) when the proctor said a cue word (e.g., stamp). Three consecutive tests were performed with related paired words in a pseudo‐random order, followed by three consecutive tests with unrelated paired words in a pseudo‐random order. Scores on the third/final test, in the unrelated tests, were used for the analyses. Because of the ceiling effect (i.e., nearly all participants scored the highest both in the first and second sessions), results of the related tests were not used for the analyses.

In both the free‐recall (AVLT) and cued‐recall (PA) tests, no feedback for memory performance was provided throughout the sessions. Participants were not instructed to perform recall tests 2 days later.

Two days later, in the post‐intervention session, participants were asked to recall an initial list of words in the free‐recall test (list A of AVLT) in the first session without any cues (2‐day delayed recall test). There were no immediate recall assessments (i.e., only delayed recall test) of AVLT in the second session.

For the cued‐recall test (PA) in the post‐intervention session, other related and unrelated lists (lists B1 and B2) were used with procedures identical to the first session. Final/third cued‐recall scores of unrelated lists in the first and second sessions were used for the analyses.

#### 3.2.3 Heart rate measures

To monitor and prevent overload during the resistance training, we measured the resting heart rate of the participants in the training group from their left wrist using the heart rate app implemented in Apple Watch (ver. 7.5, APPLE Inc.). Heart rate was measured immediately before muscle training (once) and after each set of muscle training (three times). If the heart rate was >150 beats per minute (Borg, 1982), training was discontinued. Heart rate was not measured during or after the muscle strength measurements.

#### 3.2.4 Muscle strength measurement as one repetition maximum

One repetition maximum (1RM) was measured as the maximum muscle strength required to lift the weights with both legs. A knee extension/flexion task (Weinberg et al., 2014) was performed using a leg extension bench machine (WASAI MK035) to measure the 1RM. Participants in the training group were seated in a chair with their backs erect and knees flexed to 90°. Before the measurements, they were warmed up with no weight to get used to the motion. The measurement started with a weight of 15 kg. According to the participants’ responses, the experimenter added weights of 5 or 10 kg. The maximum weight that the legs held at 60° of extension was defined as their 1RM. The 1RM measurement was performed after the memory recall tests in both sessions.

#### 3.2.5 Resistance training

During the intervention session, after a 2‐min rest following 1RM measurement, participants in the training group engaged in a knee extension/flexion task (Weinberg et al., 2014) as muscle training. The muscle training regimen consisted of three sets of eight repetitions at 80% of their 1RM. As the lightest weight was 2.5 kg, weights smaller than 2.5 kg were rounded down to calculate 80% of the 1RM which resulted in 75%–80%. A 1‐min rest was provided between sets. When participants could not complete their set, they did the maximum number per set that they were capable of. The duration of resistance training was <7 min, including rest between sets, for all participants.

#### 3.2.6 MRI scan

All images were acquired using a Philips Intera Achieva 3.0T scanner. For resting‐state functional MRI (rs‐fMRI), 34 trans‐axial gradient‐echo images (64 × 64 matrix, TR = 2000 ms, TE = 30 ms, FOV = 24 cm, 3.75‐mm slice thickness, and voxel size = 3.75 × 3.75 × 3.75 mm^3^) covering the entire brain were acquired using an echo planar sequence. For this scan, 300 functional volumes (scan duration = 10 min) were obtained while the participants were resting (opening their eyes, not moving, and not sleeping). Three‐dimensional T1‐weighted images were obtained using a magnetization‐prepared rapid gradient‐echo sequence (240 × 240 matrix, TR = 6.5 ms, TE = 3 ms, TI = 711 ms, FOV = 24 cm, 162 slices, 1.0 mm slice thickness, voxel size = 1.0 × 1.0 × 1.0 mm^3^, and scan duration = 4 min and 20 s).

#### 3.2.7 Analyses of resting‐state functional connectivity data

The data processing method was consistent with that used in our previous studies in children (Hashimoto et al., 2020; Hashimoto et al., 2021). We performed MRI data preprocessing and analysis using the Statistical Parametric Mapping (SPM12) software (Wellcome Department of Cognitive Neurology, London, UK). rsFC (signal synchrony among remote brain areas) was computed using simple correlations between the spontaneous activation levels in multiple brain areas. We did not discard any initial volumes because the MRI scanner automatically discarded the initial volumes in a non‐steady state. Prior to preprocessing, we applied the ArtRepair toolbox implemented in SPM12 to repair spike noise in slices through interpolation of before and after scans. We used the Data Processing Assistant for rs‐fMRI (http://rfmri.org/DPARSF) to preprocess the time‐series volume of each session per participant. This included realignment to the first volume, slice timing correction, T1 image co‐registration to fMRI data, segmentation of T1 images with a diffeomorphic anatomical registration through an exponentiated lie (DARTEL) algebraic registration process, normalization to the Montreal Neurological Institute (MNI) space by DARTEL, spatial smoothing (6 mm full‐width half‐maximum), detrending, and temporal filtering (0.01–0.1 Hz). After spatial smoothing, and before detrending, the ArtRepair toolbox was used to detect and repair bad volumes through interpolation. The criteria for bad volumes were (1) a 1.5% variation in the global signal intensity and (2) excessive scan‐to‐scan motion, defined as a 0.5 mm frame‐wise displacement (FD). Moreover, we used the Friston‐24 model to regress out nuisance covariates, including 6 head motion parameters, 6 head motion parameters 1 time point before, and 12 corresponding squared items. The global mean signal was not regressed because it can remove true neuronal signals and diminish the connectivity–behavior relationship (Murphy & Fox, 2017). White matter and cerebrospinal fluid signals were regressed to reduce head motion effects using an anatomical component‐based noise‐correction method (Behzadi et al., 2007; Muschelli et al., 2014) with T1 segment masks and the top five principal components. We used the exclusion criterion of mean FD >.3 mm (Power et al., 2014) to account for excessive head movement.

We selected four seed regions of interest (ROIs) from an rsFC study (Wagner et al., 2016). The left anterior hippocampus (MNI coordinates: −28, −12, −20), left posterior hippocampus (MNI coordinates: −28, −24, −12), right anterior hippocampus (MNI coordinates: 28, −12, −20), and right posterior hippocampus (MNI coordinates: 32, −22, −12), all with a 6‐mm radius sphere, were defined as seed ROIs. The anterior‐posterior hippocampal segmentation was consistent with another study that examined memory‐related activation with MNI coordinates of *y* = −21, corresponding to the appearance of the uncus of the parahippocampal gyrus (Langnes et al., 2019).

Pearson's correlations between the mean time course of each ROI and that of each voxel of the whole brain were calculated at the single‐participant level, and the correlation coefficients were transformed using Fisher *z*‐transformation.

### 3.3 Statistical analysis

Physiological and memory performance data were analyzed using SPSS Statistics software version 24 (IBM, Armonk). A statistical threshold was set to *p* < .05. Physiological changes between the first and second sessions in the training group were compared using two‐sample *t* tests. As higher memory performance in the training than control group was expected, the 2‐day delayed free recall score was compared between the training and control groups using a one‐tailed *t* test. For cued‐recall scores, final/third cued‐recall scores of unrelated lists in the first and second sessions were used for the analyses using a repeated ANOVA (two groups × two session). Spearman's rank correlations between physiological data and memory recall scores were calculated.

For comparing hippocampal FC between the sessions, we performed paired *t*‐tests with covariates of sex and mean FD of the sessions to examine the effects of muscle training on FC using SPM12 with a statistical threshold for family‐wise error (FWE) of *p* < .05 at the cluster level with uncorrected *p* < .001 at the voxel level. Further, a subtraction image (post minus pre) for each participant was made, and we also performed multiple regression analyses using memory recall scores, sex, and mean FD as covariates using SPM12. We applied a statistical threshold for FWE of *p* < .05 at the cluster level with uncorrected *p* < .001 at the voxel level.

Finally, we extracted connectivity strength from the SPM results (6‐mm sphere centered at peak coordinates) of multiple regression analyses and calculated the correlations (Spearman's correlation coefficient) between connectivity strength and memory recall scores with a statistical threshold of *p* < .05 using SPSS.

## 4 RESULTS

### 4.1 Physiological measures

Mean body weight, BMI, and muscle strength measured by 1RM in the sessions, and post‐intervention changes in the training group are shown in Table 1. There were no significant changes in body weight (*t*(29) = .39, *p* = .70) and BMI (*t*(29) = .48, *p* = .63). The 1RM significantly increased 2 days after muscle training (*t*(29) = 4.94, *p* < .001).

**TABLE 1:** Mean physiological measures in the training group.

	Pre‐intervention	Post‐intervention	Change	
Body weight (SD)	56.1 (7.7)	56.2 (7.8)	0.1 (1.3)	kg
Body mass index (SD)	20.2 (1.8)	20.3 (1.9)	0.0 (0.5)	
1RM (SD)	34.2 (11.1)	38.2 (11.3)	4.0 (4.4)^***^	kg

Abbreviations: 1RM, one repetition maximum; SD, standard deviation.

*** *p* < .001.

Men showed higher body weight (59.0 vs. 51.9, *t*(28) = 2.74, *p* = .01) and 1RM (39.2 vs. 26.7, *t*(26) = 4.10, *p* = .001) than women pre‐intervention. No significant sex differences were observed in pre‐intervention BMI (20.3 vs. 20.1, *t*(28) = .41, *p* = .68). No sex differences were detected in body weight change (*t*(28) = .04, *p* = .97), BMI change (*t*(28) = .06, *p* = .95), or 1RM (*t*(28) = .17, *p* = .87).

The mean (standard deviation) heart rate before muscle training was 77.2 (12.5), and the mean maximum heart rate during or immediately after muscle training was 106.4 (11.2). None of the participants showed signs of overload (>150 beats per minute).

### 4.2 Memory performances

#### 4.2.1 Cued‐recall test

**FIGURE 2:**
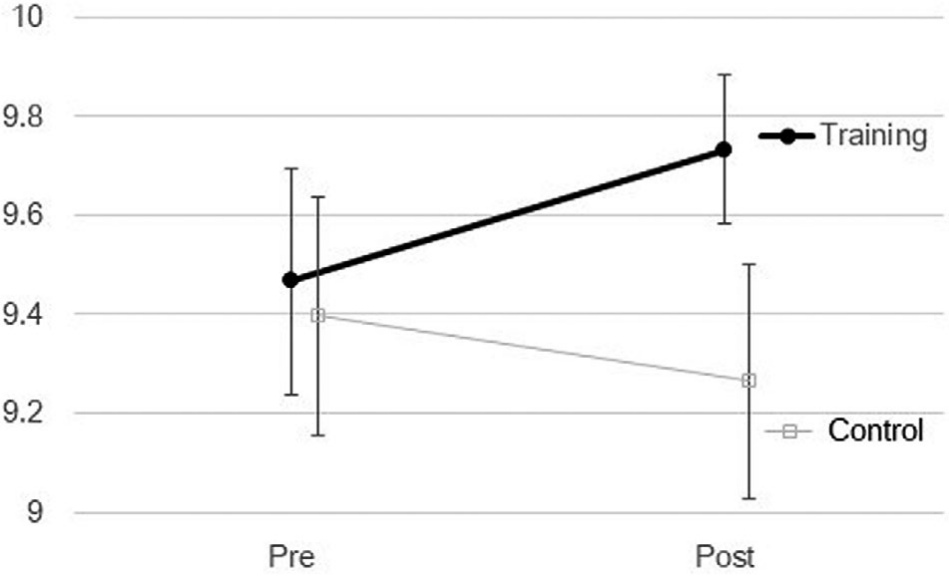
Cued‐recall test scores before and 2 days after muscle training. No significant differences in groups and sessions, but significant interaction effect was observed in cued‐recall scores of unrelated word‐pairs. The effect of resistance training on cued‐recall scores was different from that in waiting/control at 2 days after resistance training. Error bars show standard error.

Many participants scored the highest in cued‐recall tests, and less individual differences were observed. In unrelated word pair recall, a repeated ANOVA revealed no statistically significant effects of group (F[1,58] = 0.79, *p* = .38) nor session (F[1,58] = 0.80, *p* = .37) in cued‐recall performance, but the interaction (F[1,58] = 7.20, *p* = .01) was observed as shown in Figure 2. Mean score changes were +0.27 in the training group and −0.13 in the control group. Although no significant group differences were observed, these results could suggest effects of resistance training on the cued‐recall performance.

#### 4.2.2 Free recall test

Mean free‐recall scores in the post‐test in the training group were significantly higher than those in the control group (9.5 vs. 8.0, *t*(58) = 1.74, *p* = .04, one‐tailed). Of the 15 words learned in the first session, approximately 63% words could be recalled without any cues after a 2‐day delay in the training group, compared to 53% in the control group.

### 4.3 Correlations between physical measures and memory performance

The proportions of muscle strength (1RM), BMI, and cued recall scores in the two sessions (post/pre as Δ) were used. The maximum heart rate during resistance training in the first session and the free recall scores in the post‐intervention session were also used. Spearman's rank correlation coefficients showed no significant correlations and only weak relationships between the physical measures and memory performance (Table 2).

**TABLE 2:** Correlations between physical measures and memory performance in the training group.

	1	2	3	4
1. Δ1RM				
2. ΔBMI	0.19			
3. Heart rate max	−0.11	0.21		
4. ΔCued‐recall	0.20	0.19	−0.25	
5. Free‐recall	0.09	0.06	0.11	0.27

*Note*: Spearman's rank correlation coefficients.

Abbreviations: 1RM, one repetition maximum; BMI, body mass index.

### 4.4 Changes in hippocampal connectivity 2 days after acute muscle training

No significant differences in head movement parameter (FD) between the two sessions were detected with a paired *t*‐test (0.16 vs. 0.16 for the first and second sessions, respectively (*t*(29) = .40, *p* = .69). None of the participants showed excessive head movement (FD > .3).

**FIGURE 3:**
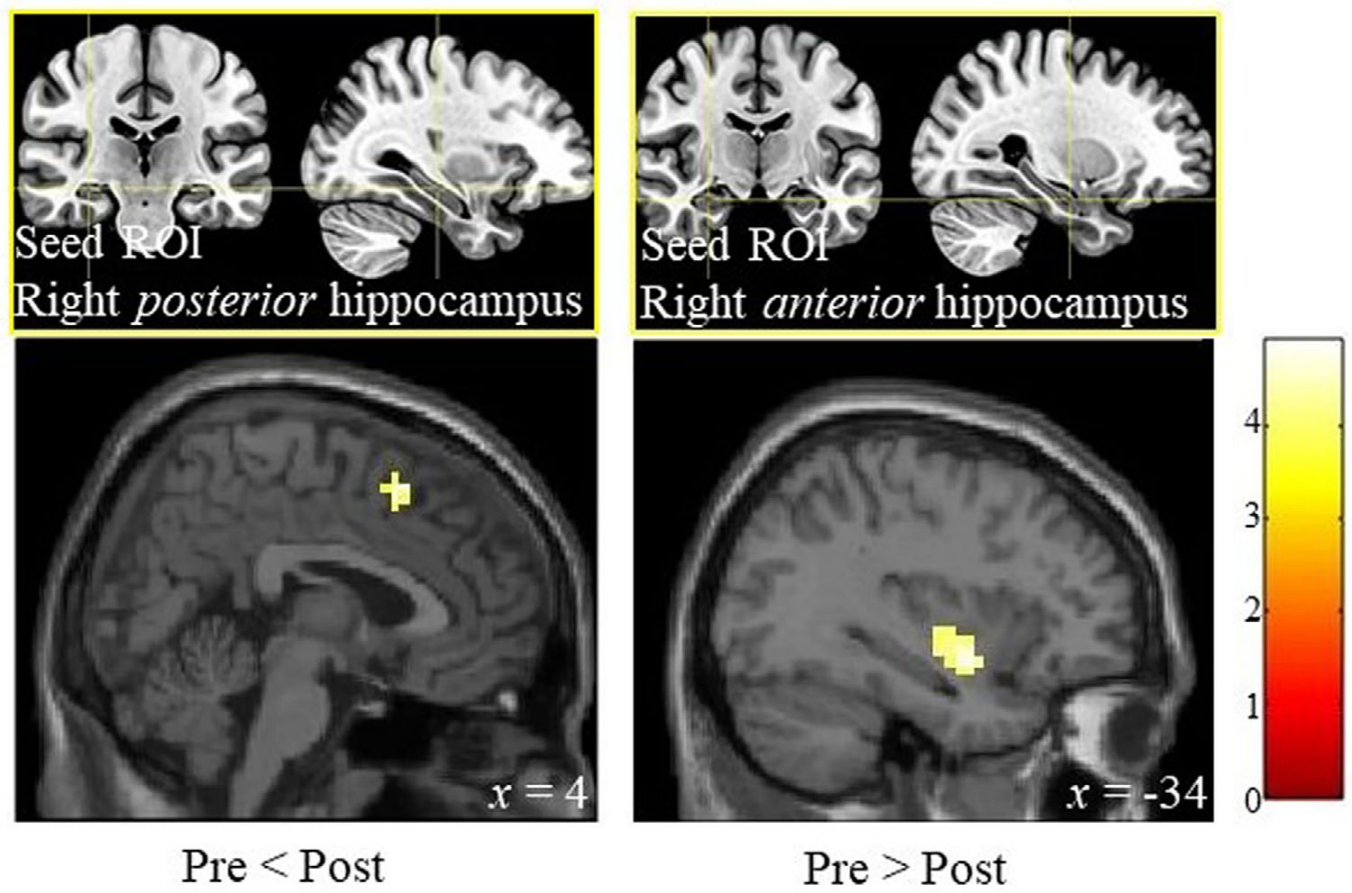
Hippocampal connectivity 2 days after acute muscle training. Paired *t*‐tests revealed both enhanced and reduced hippocampal connectivity in the post‐test 2 days after resistance training compared with pre‐intervention state. Enhanced connectivity between the right posterior hippocampus (seed region of interest [ROI]) and the medial frontal cortex (left panel), and reduced connectivity between the right anterior hippocampus (seed ROI) and the left posterior insula are observed (right panel). Family‐wise error–corrected *p* < .05 at cluster level and uncorrected *p* < .001 at voxel level. Sex and mean head movement parameter of the sessions are used as covariates of no interest. The color bar shows *z* value, and *x* indicates Montreal Neurological Institute coordinates..

Paired *t*‐tests revealed significant differences in hippocampal connectivity between pre‐ and post‐interventions independent of memory performance (Table 3, FWE of *p* < .05 at the cluster level with uncorrected *p* < .001 at the voxel level). Greater connectivity was observed between the right posterior hippocampus and medial frontal cortex post‐ than pre‐intervention (Figure 3, left panel). Reduced connectivity between the right anterior hippocampus and the left posterior insula was observed post‐intervention compared with pre‐intervention (Figure 3, left panel).

**TABLE 3:** Changes in hippocampal connectivity 2 days after muscle training.

Hippocampal				Coordinates		
Seed ROIs	Brain area	Voxels	*z* Value	*x*	*y*	*z*
Enhanced (post > pre)						
Left *anterior*	–					
Left *posterior*	–					
Right *anterior*	–					
Right *posterior*	Medial frontal cortex	39	4.24	4	15	53
Reduced (pre > post)						
Left *anterior*	–					
Left *posterior*	–					
Right *anterior*	–					
Right *posterior*	Left posterior insula	69	4.13	−34	0	−11

### 4.5 Changes in hippocampal connectivity by acute resistance training in association with memory performance

As only few changes between sessions were observed in word‐pair recall scores, the relationships between these and hippocampal connectivity changes were not examined.

**FIGURE 4:**
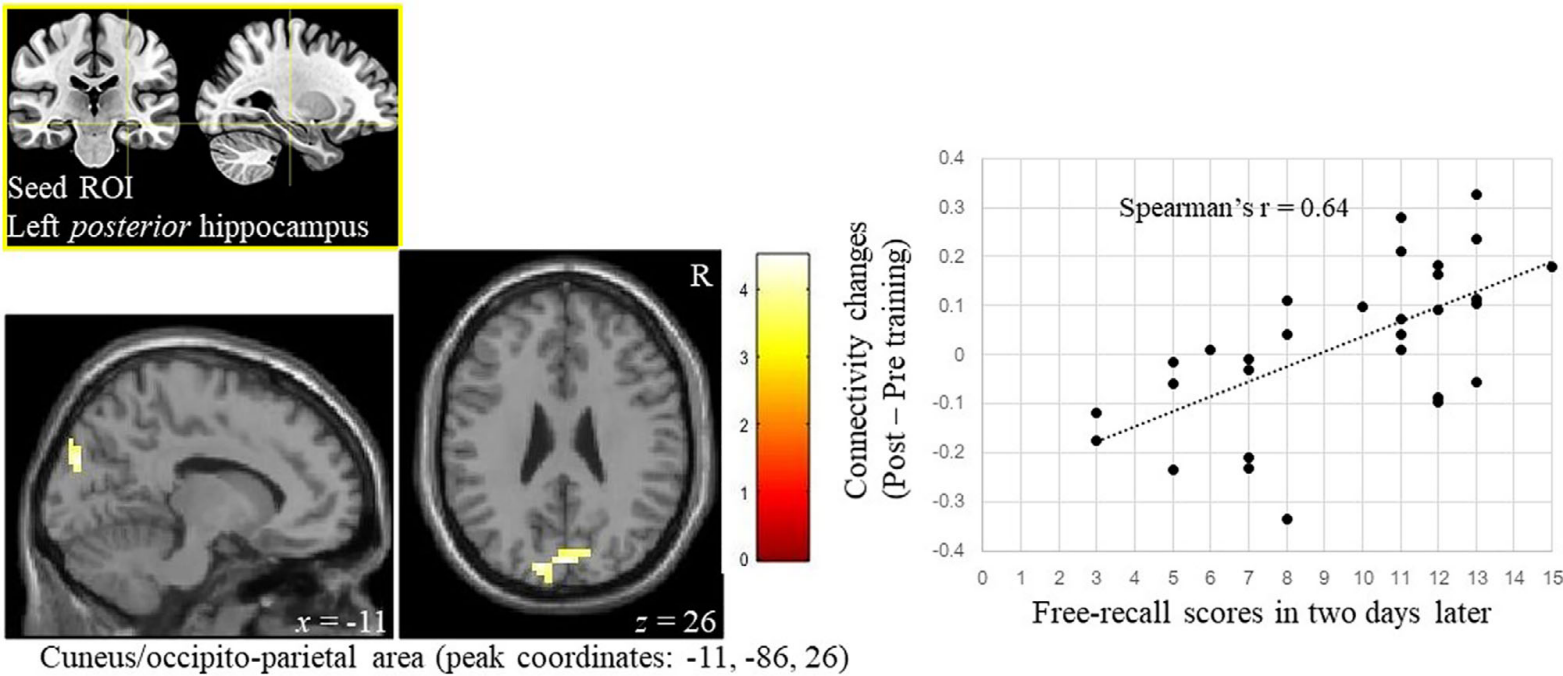
Enhanced hippocampal connectivity 2 days after muscle training in association with free‐recall scores. Enhanced connectivity between the left posterior hippocampus (seed region of interest [ROI]) and the cuneus to parieto‐occipital area was positively associated with free‐recall scores in the post‐test compared with pre‐intervention. The scatter plot shows individual recall scores and connectivity changes. Family‐wise error–corrected *p* < .05 at cluster level and uncorrected *p* < .001 at voxel level. Sex and mean head movement parameter of the sessions are used as covariates of no interest. The color bar shows *z* value. *R* denotes right, whereas *x* and *z* indicate Montreal Neurological Institute (MNI) coordinates.

Multiple regression analyses revealed free‐recall scores correlated with connectivity strength changes between the left posterior hippocampus and cuneus post‐intervention (Spearman's *r* = .64, *p* < .001, Figure 4). The cluster extended to the right hemisphere (voxels: 56, *z*‐value = 3.83, peak coordinates: [−11, −86, 26]). The right hippocampal connectivity changed after acute muscle training (independent of memory performance in Figure 3 and Table 3) and showed no significant correlations with free‐recall scores.

## 5 DISCUSSION

A single bout of high‐intensity resistance training affected memory and hippocampal connectivity after only 2 days in healthy young adults. The findings provide evidence that a single bout of resistance training could be effective in improving memory and brain function after a few days.

Improvements in muscle strength (1RM) and cued recall performance were observed 2 days after resistance training. Meanwhile, no apparent correlation between physical condition and memory performance was detected. These results suggest that the effects of resistance training on the body (muscle) could be partially dissociated from those on memory with a 2‐day delay. It could be possible that improvement in 1RM was caused by habituation of settings (i.e., the leg extension task and machine), not by increased muscle strength. The timing of training (before or after encoding) could be important for detecting the relationship between acute resistance training and memory performance (Loprinzi et al., 2020), and our timing of training might not be optimal. Alternatively, a longer delay period and/or more training sessions may be required to determine a clear relationship between resistance training and memory. Furthermore, the optimal time delays between resistance training and effects of rsFC were unknown.

Effects of muscle training on right hippocampal connectivity over 2 days, independent of memory performance, were detected. Enhanced connectivity between the right posterior hippocampus and medial prefrontal cortex was consistent with effects of higher cardiorespiratory fitness (Stillman et al., 2018). Resistance training might have positive effects on cardiorespiratory fitness (Feiereisen et al., 2007; Vonbank et al., 2012). In addition, reduced connectivity in the right anterior hippocampal network might be related to a shift in attentional focus (Stillman et al., 2018). Increased right hippocampal connectivity induced by electroconvulsive therapy has been correlated with a reduction in depressive symptoms (Abbott et al., 2014). Besides mood, sleep efficiency and duration can be improved by exercising (Stillman et al., 2020). These possible psychosocial improvements might have been induced by our resistance training, which was associated with changes in right hippocampal connectivity. Moreover, the longitudinal axis of the hippocampus shows an anterior‐to‐posterior gradient of coarse‐to‐fine spatiotemporal representations (Brunec et al., 2018). The enhanced posterior and reduced anterior hippocampal connectivity after resistance training in this study might be related to finer and more detailed spatiotemporal processing, which could be beneficial to cognitive and affective functions, including memory.

Free recall scores after 2 days were positively correlated with connectivity changes between the left posterior hippocampus and the cuneus/occipito‐parietal area 2 days after resistance training. Our results replicate a recent finding that brief and mild exercise induces memory improvement and enhances the posterior medial temporal network (Suwabe et al., 2018). The connectivity between the left posterior hippocampus and the cuneus/occipito‐parietal area involved in visuospatial processing (Kravitz et al., 2011) could be associated with free recall scores with a 2‐day delay. Familiarity or fluency of words can be associated with connectivity between the posterior hippocampus and the cuneus (Gomes et al., 2019). Increased connectivity in these regions might enhance memory, even for words learned through auditory verbal inputs. Moreover, these results are consistent with the functional gradients of the hippocampus (Robinson et al., 2016): the posterior hippocampus for fine spatiotemporal representations or recollection (Brunec et al., 2018; Dalton et al., 2019; Persson et al., 2018; Poppenk & Moscovitch, 2011), and left hippocampal involvement in contextual, relational, and specific memory (Burgess et al., 2002; Prince et al., 2005). There are controversies regarding the left/right laterality of hippocampal function, and the right hippocampus may be associated with the cued recall of word pairs (Hashimoto et al., 2011). Connectivity between the posterior hippocampus and parahippocampal gyrus has been associated with the recollection of contextual details (Ranganath & Ritchey, 2012). Although no direct relationship between resistance training and posterior hippocampal network changes (post‐intervention differences independent of memory performance) was detected, improved memory was associated with the left posterior medial temporal network after resistance training.

## 6 CONCLUSIONS

A single bout, short duration, and high‐intensity resistance training could improve memory and hippocampal connectivity after a few days in young adults. Simultaneously, there were individual differences in the beneficial effects of resistance training, and some participants did not show enhanced hippocampal connectivity. The quick effect of resistance training on memory could be detected, whereas more time might be required for the improvement of neuroplastic mechanisms. Resistance training might potentially benefit those who need brief exercising.

## AUTHOR CONTRIBUTIONS

**Teruo Hashimoto**: Conceptualization; methodology; investigation; formal analysis; writing—original draft; writing—review and editing; data curation. **Rikimasa Hotta**: Data curation; investigation; formal analysis; writing—review and editing; methodology; conceptualization; writing—original draft. **Ryuta Kawashima**: Writing—review and editing; supervision.

## CONFLICT OF INTEREST STATEMENT

The authors declare no conflicts of interest.

## FUNDING INFORMATION

No funding was received for conducting this study.

### PEER REVIEW

The peer review history for this article is available at https://publons.com/publon/10.1002/brb3.3436.

## Data Availability

The data that support the findings of this study are available from the corresponding author upon reasonable request.
